# Modulating oxidative stress: a reliable strategy for coping with community-acquired pneumonia in older adults

**DOI:** 10.3389/fmed.2025.1549658

**Published:** 2025-03-26

**Authors:** Weixu Mao, Xuanjun Liu, Senji Fan, Ruibin Zhang, Miao Liu, Shunqiong Xiao

**Affiliations:** ^1^Department of Respiratory Medicine, The Affiliated Yongchuan Traditional Chinese Medicine Hospital of Chongqing Medical University, Chongqing, China; ^2^Department of General Surgery, The Affiliated Yongchuan Hospital of Chongqing Medical University, Chongqing, China

**Keywords:** community-acquired pneumonia, older adults, oxidative stress, inflammation, aging

## Abstract

Community-acquired pneumonia (CAP) remains one of the leading respiratory diseases worldwide. With the aging of the global population, the morbidity, criticality and mortality rates of CAP in older adults remain high every year. Modulating the signaling pathways that cause the inflammatory response and improve the immune function of patients has become the focus of reducing inflammatory damage in the lungs, especially CAP in older adults. As an important factor that causes the inflammatory response of CAP and affects the immune status of the body, oxidative stress plays an important role in the occurrence, development and treatment of CAP. Furthermore, in older adults with CAP, oxidative stress is closely associated with immune senescence, sarcopenia, frailty, aging, multimorbidity, and polypharmacy. Therefore, multiple perspectives combined with the disease characteristics of older adults with CAP were reviewed to clarify the research progress and application value of modulating oxidative stress in older adults with CAP. Clearly, there is no doubt that targeted modulation of oxidative stress benefits CAP in older adults. However, many challenges and unknowns concerning how to modulate oxidative stress for further practical clinical applications exist, and more targeted research is needed. Moreover, the limitations and challenges of modulating oxidative stress are analyzed with the aim of providing references and ideas for future clinical treatment or further research in older adults with CAP.

## 1 Introduction

*Community-acquired pneumonia* (CAP) refers to the infectious inflammation of the lung parenchyma (including the alveolar wall, i.e., pulmonary interstitium in general) acquired outside of hospitals, including pneumonia caused by pathogens with proven latency (1). CAP is one of the most common acute respiratory infections worldwide and can be caused by bacteria, viruses, fungi, or other pathogens alone or in combination. The common causative agents of CAP may vary from country to country, but the older adults and children have always been the primary groups of victims (2, 3). In particular, the global pandemic of *coronavirus disease 2019* (COVID-19) has put unprecedented pressure on healthcare worldwide and incurred non-negligible medical costs (4). Oxidative stress, as one of the pathogenic mechanisms of CAP, has been widely demonstrated to be closely linked to inflammation and the immune response (5–7). In recent years, most studies related to the treatment of CAP have focused on reducing inflammation and oxidative damage in the lungs. However, immunosenescence, sarcopenia, frailty, multimorbidity, polypharmacy, and other aging-related disease risk factors are also present in older CAP patients. The impact of these factors on the development, treatment and prognosis of CAP in the older individuals and their relationship with oxidative stress are not sufficiently clear. In addition, the potential value and advantages of modulating oxidative stress compared with the traditional preventive and therapeutic modalities of CAP have rarely been mentioned. Therefore, in conjunction with the above unique disease manifestations present in the older population, this review on more links between oxidative stress and CAP in older adults aims to further clarify the possible benefits of modulating oxidative stress in older adults with CAP patients, with the goal of providing a reference for the treatment of CAP in older adults and related research in the future.

## 2 Occurrence of CAP and activation of the immune response

The lung is a complex microbial ecosystem. The microbes are in complex relationships with each other and with the host, in a state of mutual adaptation and dynamic interaction. Currently, it is recognized that most pathogens invade through the respiratory tract, and infection occurs when host defenses are compromised and/or when the host is exposed to highly virulent microorganisms or large amounts of inoculum. After entering the nasopharynx, pathogens escape recognition and clearance from host immune cells by mimicking the host molecular structure or altering their own antigens, escaping the mucus and adhering to the upper respiratory epithelium (8, 9).

**Figure 1:**
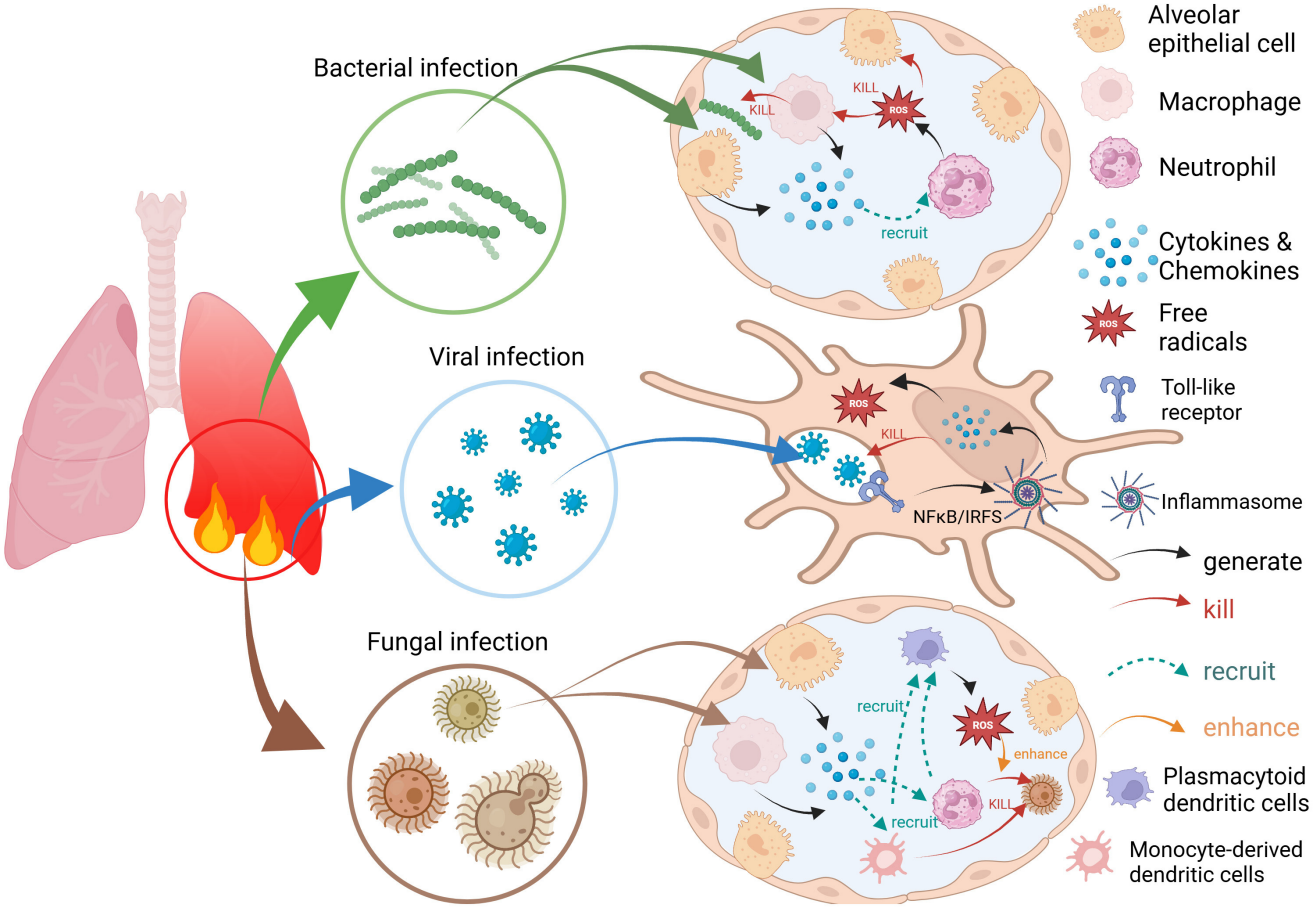
Bacterial, viral, and fungal infections trigger immune and inflammatory responses and the process of ROS generation.

The manner in which bacteria, viruses and fungi induce an immune response after invasion is somewhat different (Figure 1). When bacteria invade the alveoli and then multiply, they are sensed and recognized by *polymorphonuclear leukocytes* (neutrophils), which in turn elicits the body's innate immune response. Bacteria interact with alveolar cells, such as alveolar epithelial cells and macrophages, which secrete cytokines and neutrophil chemokines and subsequently recruit additional immune cells from the pulmonary circulation to the site of infection. These immune cells produce proteases, *reactive oxygen species* (ROS) and *reactive nitrogen species* (RNS) and act on infected cells to induce necrotic cell death (10). For viral antigens, the adaptive immune response is activated mainly by the encounter of viral particles with antigen-presenting cells or B-cell receptors and further induces a variety of immune cell interactions and the production of cytokines, chemokines, etc., to inhibit viral replication and transmission and protect the host from viral attack (11, 12). Certainly, viruses also evade immune cell recognition and antiviral responses by increasing their affinity for target cells, inhibiting the recognition of relevant receptors and disrupting immune signaling pathways. It can even provoke the immune system to attack its own tissues in the form of inducing autoimmune and autoinflammatory processes that allow it to survive in the host (13). For fungi, within the respiratory tree, inhaled fungal cells are opsonized with soluble pattern recognition receptors, surfactants, complement, and antibodies. Mainly cells such as neutrophils, monocytes, monocyte-derived dendritic cells, *plasmacytoid dendritic cells* (pDCs), and mediators such as cytokines, neutrophil chemotaxis, and interferon are involved in the whole immune process. Among these, pDCs do not bind conidia but enhance the oxidative burst to boost conidial killing in neutrophils (14).

Certainly, there are many other disease-causing pathogens in CAP, but the same is true for the various immune responses of the organism to pathogens, which are usually beneficial to the host (15–17). However, some studies have noted that overzealous activation of the innate immune system can lead to immunopathology, which may result in poor outcomes and even contribute to secondary bacterial infections in the lower respiratory tract, which can lead to pneumonia (18–20). The occurrence of such an excessive immune response may be due to the uncontrolled production of inflammatory cytokines, which trigger the release of large amounts of inflammatory mediators and excessive production of ROS, which exacerbates or prolongs the stimulation and directly or indirectly leads to cell and tissue death and widespread lung injury, thus increasing the burden of CAP (21). Thus, there is still a delicate balance that needs to be regulated between appropriate immunoprotection and excessive pathologic immunity.

The new idea holds that the various microorganisms in the lungs have a complex relationship with the host and with each other that is analogous to an ‘adaptive island', where they maintain a dynamic equilibrium and constraints (22). On the basis of this theory, regardless of whether a new pathogen invades, disrupting the balance of existing microorganisms can lead to lung infections. The immune and inflammatory responses of an organism are measured to maintain this balance. Although the whole process is provoked by the pathogen, preinfectious susceptibility to pneumonia, the host's own characteristics, and host–pathogen interactions also play a catalytic role in the onset of CAP and the activation of the immune response (23, 24).

Compared with healthy young adults, it is commonly agreed that older adults have characteristic elevated serum levels of proinflammatory markers (25). During aging, the levels of inflammatory factors and serum proinflammatory markers increase significantly, even in older adults without chronic diseases (26). This persistent inflammation, also known as low-grade inflammation, drives the onset of age-related diseases and the exacerbation of chronic conditions such that older adults are more likely to have significant lung inflammation and lung tissue damage (27, 28). In short, multiple components, such as pathogenic microorganisms, inflammatory responses, and immune resistance, are combined to determine whether CAP occurs and how it progresses or subsides. And the impacts on older adult patients are particularly significant and complex.

## 3 Overview of oxidative stress

Oxidation and antioxidation are two systems in the body that are interdependent but also mutually restrictive, and oxidative stress is the result of a disruption of the pro-oxidant–antioxidant balance and the consequent accumulation of ROS (29). ROS are highly reactive active molecules produced by the interaction of oxygen with certain elements; they hold at least one unpaired electron in their outer shell and are therefore extremely unstable (30). ROS can either originate from outside the body or be produced by certain oxidizing enzymes in the body. However, whether endogenous or exogenous, major cellular macromolecules such as nucleic acids, lipids and proteins can be oxidatively modified under the effect of ROS, which can lead to structural and functional alterations in cells (31). Therefore, ROS can be involved in both physiological apoptosis and pathological cell death.

Fortunately, the innate cellular antioxidant system also blocks overly intense inflammatory responses and prevents excessive ROS accumulation, thus limiting damage and inhibiting disease (32). Antioxidant defenses can be divided into non-enzymatic and enzymatic antioxidants. Non-enzymatic antioxidants include those naturally produced by cells, such as *glutathione* (GSH), *coenzyme Q10* (CoQ10), and melatonin, and non-naturally produced antioxidants, such as *vitamin C* (VitC), *vitamin E* (VitE) and flavonoids (33). They are characterized by the ability to act directly and quickly inactivate free radicals and oxidants (34). GSH is an essential and important non-enzymatic antioxidant in mammalian cells. It is not only used directly as an antioxidant to protect cells from free radicals and pro-oxidants but also as a cofactor for antioxidant and detoxification enzymes such as *glutathione peroxidase* (GPX), glutathione S-transferase and glyoxalase (35). The main antioxidant enzymes include *catalase* (CAT), *superoxide dismutase* (SOD), GPX, etc. O_2_ is converted to H_2_O_2_ by SOD, which is then broken down by CAT into O_2_ and H_2_O, thereby preventing the generation of hydroxyl radicals. GPX converts peroxides and hydroxyl radicals to non-toxic forms by oxidizing GSH to glutathione disulfide, which is then reduced to GSH by glutathione reductase (36, 37).

**Figure 2:**
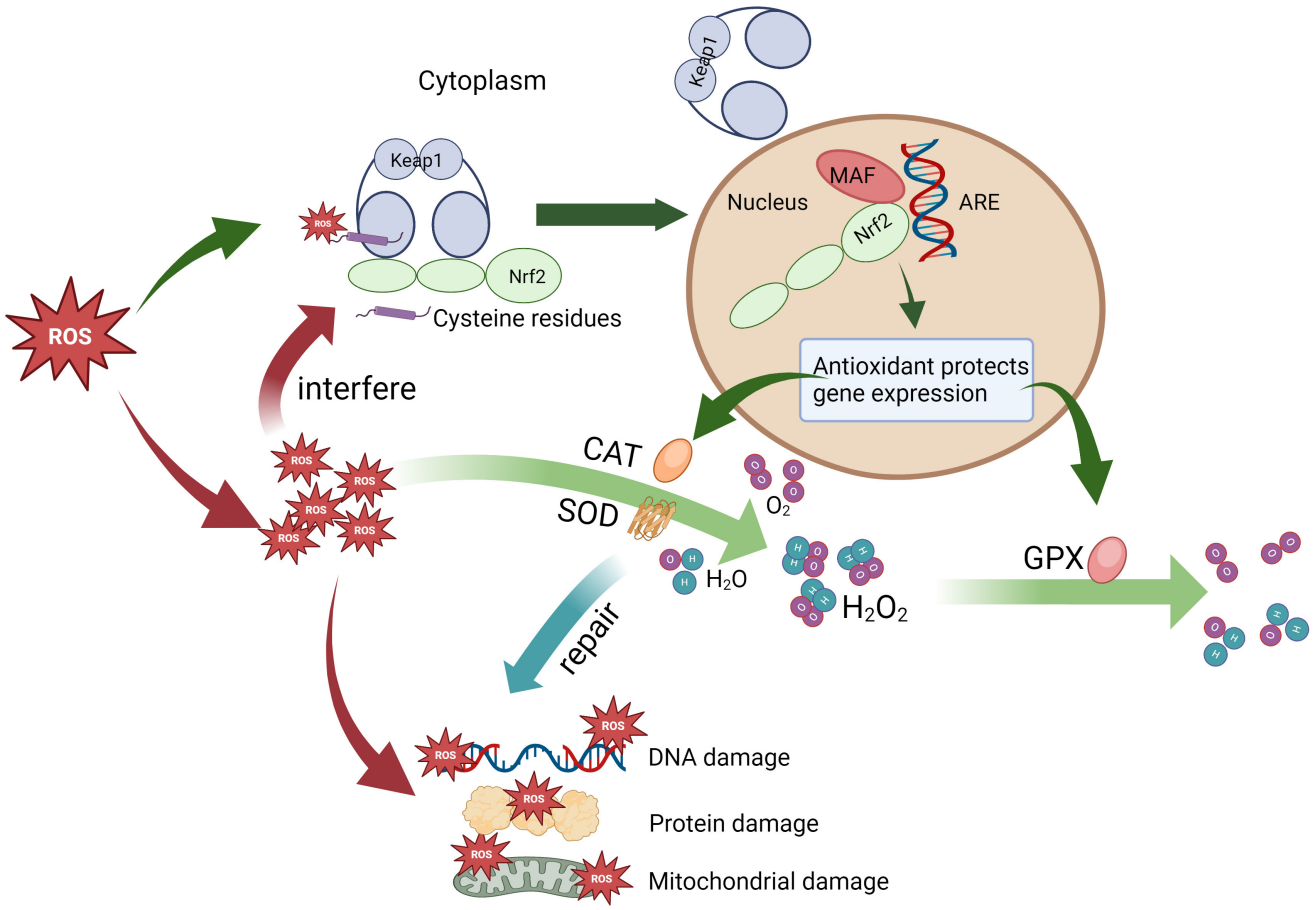
The antioxidant stress response mediated by the Nrf2/Keap1 signaling pathway.

The antioxidant response is modulated mainly by the Nrf2/Keap1 system (Figure 2). Nrf2 (*NF-E2-related factor 2*) is a transcription factor, whereas Keap1 (*kelch-like ECH-associated protein 1*) is an Nrf2 repressor. Under physiological conditions, Keap1 acts as a negative regulator of Nrf2, locking Nrf2 in the cytoplasm and preventing it from entering the nucleus. On the other hand, Keap1 mediates the ubiquitination-mediated degradation of Nrf2 to maintain intracellular Nrf2 homeostasis. When stimulated by ROS, the cysteine residues on Keap1 are modified by ROS, and Nrf2 dissociates from Keap1. The activated Nrf2 enters the nucleus and binds to the Maf protein to form a heterodimer. Binding to *antioxidant response elements* (AREs) induces the transcription of genes regulated by AREs, which initiates the expression of genes encoding cytoprotective enzymes, such as antioxidative stress proteins, especially antioxidant enzymes (38). However, upon exposure to oxidative stress, Keap1 loses the ability to ubiquitinate Nrf2, leading to disruption of the redox balance, which may induce damage to cellular mitochondria, proteins, DNA, etc. (39).

The antioxidant response is also modulated by signaling pathways mediated by *adenosine 5'-monophosphate-activated protein kinase* (AMPK). AMPK is a serine/threonine kinase expressed in all eukaryotic tissues and organs, playing a key role in *adenosine triphosphate* (ATP) synthesis (40). As a central cellular energy sensor, AMPK becomes activated during metabolic perturbations including oxidative stress and inflammatory conditions (40–42). Previous study has shown that AMPK plays cytoprotective roles in glucose and lipid metabolism and protein synthesis (43). AMPK activation also preserves mitochondrial function by stabilizing redox homeostasis and membrane potential, thereby suppressing apoptosis (43). Key AMPK-dependent transcription factors, including *Forkhead box O3a* (FoxO3a), Nrf2, and Sirt1, coordinate the expression of antioxidant defense genes (44–46). Under hypoxia, mitochondria induce hypoxia-inducible factor 1α (HIF-1α) expression, a process accompanied by ROS production (47). To modulate cellular energy homeostasis, AMPK enhances FoxO3a transcription (48). Induced FoxO3a mitigates increased ROS levels in hypoxic cells by decreasing HIF-1α accumulation, exerting anti-oxidative stress effects, and altering the cellular hypoxic response (49). AMPK activation directly causes Nrf2 to migrate and aggregate into the nucleus and indirectly increases nuclear Nrf2 levels, thereby promoting antioxidant enzyme gene expression to combat oxidative damage (32). Furthermore, AMPK increases Sirt1 activity in cells with oxidative stress-induced senescence (50, 51). The sirtuin protein family, particularly Sirt1, acts as a key epigenetic regulator of aging, exerting protective effects against sarcopenia and frailty in older adults and promoting longevity (52). Sirt1-mediated deacetylation of *peroxisome proliferator-activated receptor* γ *coactivator-1*α (PGC-1α) enhances mitochondrial stabilization in skeletal muscle, as shown in *in vitro* and *in vivo* models (53). Meanwhile, Sirt1 exerts anti-oxidative stress effects by enhancing the expression of antioxidants such as MnSOD, CAT, and GPX through deacetylation and activation of pathways like PGC-1α and FoxO3a (52). Conversely, Sirt1 inhibition increases ROS production (54). In conclusion, cellular defense against ROS overload involves coordinated mechanisms, with antioxidant enzyme systems playing a pivotal role in ROS scavenging. Furthermore, antioxidant enzymes have repair mechanisms for free radical-induced damage. It can repair damaged DNA and proteins, fight against oxidized lipids, stop chain propagation of peroxyl lipid radicals, and repair damaged cell membranes and molecules (55).

## 4 Relationship between oxidative stress and CAP

**Figure 3:**
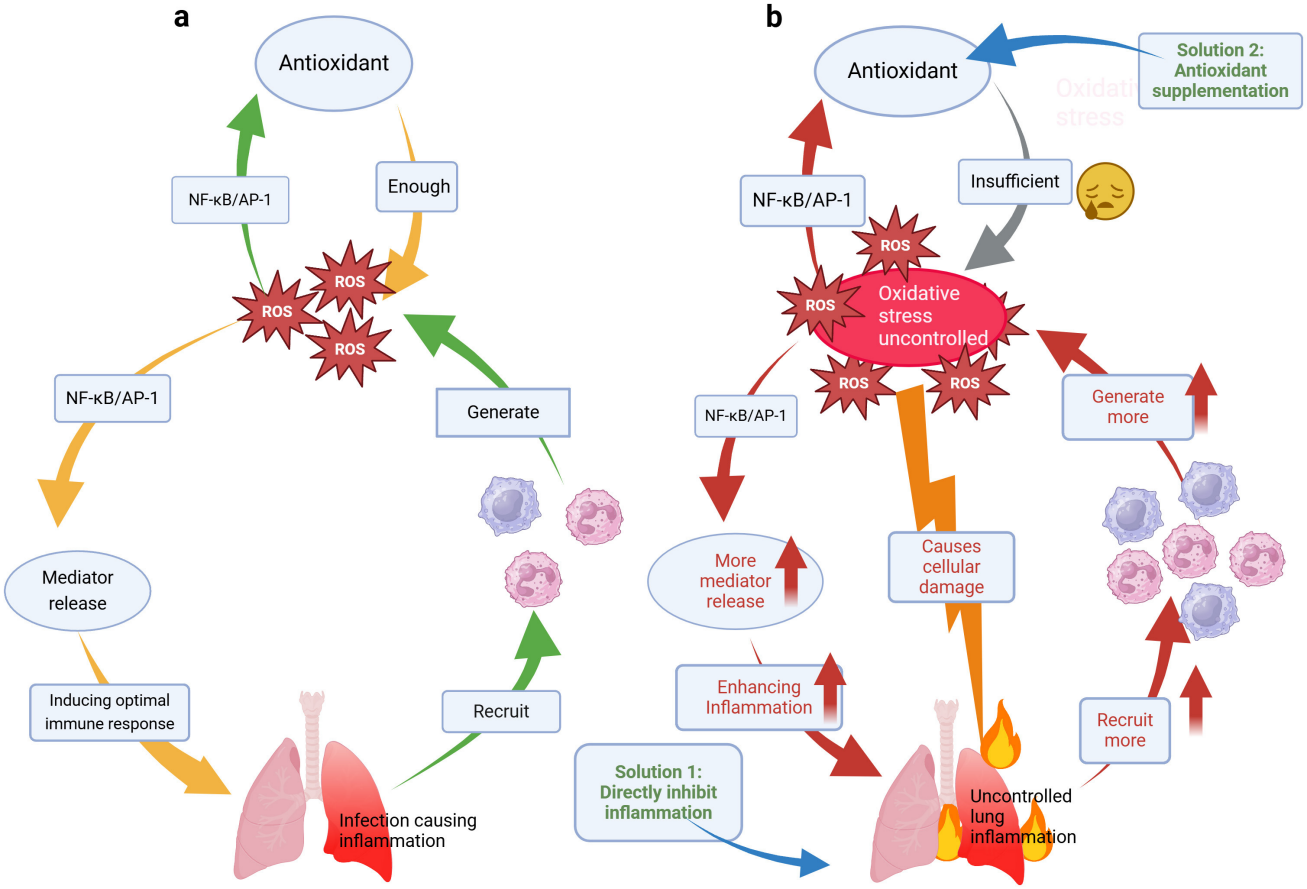
**(a)** Mechanistic models of the reciprocal modulation of the inflammatory response and oxidative stress. **(b)** Vicious cycle of uncontrolled oxidative stress and an uncontrolled inflammatory response and strategies to address this cycle.

Oxidative stress is an important component of the innate immune system and is part of the defense mechanism against pathogens (56). On the one hand, oxidative stress is considered to be one of the pathogenic mechanisms of CAP and is closely related to inflammation (57). Numerous studies have shown that oxidative stress is greater in patients suffering from CAP than in healthy volunteers (6, 58, 59). Indeed, after the onset of pneumonia, immune cells respond by releasing ROS and inflammatory factors at the site of infection. For example, macrophages are major contributors to the immune response. Upon activation, macrophage TXNIP protein induces ROS production, causing *endoplasmic reticulum* (ER) stress. This, in turn, triggers cellular signaling processes, including redox homeostasis, inflammation, and immune responses (60). During ER stress, *stimulator of interferon genes* (STING), a transmembrane protein predominantly located in the ER, responds to stress signals and promotes inflammasome activation, such as NLRP3, via the STING/*TANK-binding kinase 1* (TBK1) pathway, leading to tissue inflammation (61). Neutrophils, another key cell type involved in killing bacteria and other microorganisms, activate *NADPH oxidase* (NOX2) on their cell membranes upon stimulation. This generates large amounts of superoxide and ROS, which react with pathogens in phagosomes and trigger an inflammatory response (62). In contrast, lung cells respond to oxidative responses by activating *nuclear factor kappa-light-chain-enhancer of activated B cells* (NF-κB) and *activating protein-1* (AP-1), which inhibits oxidative stress while also stimulating the release of cytokines such as *tumor necrosis factor*-α (TNF-α) (57, 63). In this way, while maintaining the balance of the redox system, lung cells are able to generate a certain inflammatory response to trigger an optimal immune response. A review states that ROS generated by immune and inflammatory responses in tissues and organs act as potent killers of various pathogens, and controlled accumulation of ROS serves as an effective weapon against pathogen invasion (32). For example, in neutrophils, pathogens are captured and internalized via phagocytosis, forming membrane-enclosed phagosomes (64). Within these phagosomes, pathogens are exposed to bactericidal peptides, destructive enzymes, and ROS, leading to significant degradation (62). However, once uncontrolled oxidative stress occurs and cytokines are overproduced, not only can the inflammatory state of the lungs be exacerbated, but more ROS will continue to be produced, worsening the situation (Figure 3).

Notably, if the homeostasis of the redox system is persistently disrupted, it also weakens the innate and acquired immune response, exacerbates bacterial and viral infections, causes cellular and tissue damage, and drives the progression of CAP. Specific modes of damage include affecting the CD4+/CD8+ T-cell ratio, impairing natural killer cell function and neutrophil migration/phagocytosis, and reducing the stability of the macrophage cytoskeleton to affect phagocytosis (32). At the molecular level, excess ROS can react rapidly and non-specifically with chemical groups in DNA, lipids and proteins, leading to cellular metabolic disorders, loss of function and abnormal apoptosis (65). If a vicious cycle of uncontrolled oxidative stress and an uncontrolled inflammatory response further develops, more trauma to cells and tissues can occur, leading to a poor CAP outcome. For example, when a respiratory viral infection is combined with invasive fungal disease, cytokines attract more immune cells, which in turn produce more cytokines, creating an inflammatory cycle that damages lung tissues and causes the formation of fibrin and scar tissue, which allows fluids to seep into and fill the lung cavities, leading to respiratory failure (66). However, causing oxidative stress is not always detrimental. An early review revealed that oxidative stress is associated with persistent bacterial infections and antibiotic resistance. Pathogenic bacteria may undergo physiological changes by adapting to oxidative stress, either by promoting bacterial survival during antibiotic exposure or by influencing the oxidative stress response mechanism, thereby reducing the effective intracellular concentration of the antibiotic and allowing the bacteria to survive (67).

Beyond genetically resistant bacteria, persisters, which are genetically susceptible cells that survive antibiotic doses that kill the rest of the clonal population (68). These persisters are *non-growing* (NG) bacteria that coexist with their growing counterparts but are transiently insensitive to antibiotics (69). They are thought to resume growth after antibiotic treatment ceases, leading to chronic infections, recurrences, and subsequent treatment failure (70, 71). Altered adaptation to oxidative stress is a major driver of antibiotic resistance in these persisters (72, 73). A recent research has shown that *in vitro* treatment of stationary-phase *Staphylococcus aureus* (*S. aureus*) with *fluoroquinolones* (FQ) enhances FQ lethality by stimulating ROS production in cells (74). Thus, enhancing oxidative stress might be valuable in cases of persistent bacterial infections and/or antibiotic resistance. However, the role of ROS in controlling bacterial infections and mediating bacterial responses to antibiotics is complex. Studies indicate that in the case of the major Gram-positive pathogen, *S. aureus*, ROS are inhibitory to antibiotic mediated killing and their generation by the host increases *S. aureus* antibiotic tolerance during infection (75, 76). Another study found that after engulfment by macrophages, sustained production of RNS by host cells locks persisters in a NG state via tricarboxylic acid-cycle intoxication, thereby poisoning bacterial respiration. Chemical inhibition of RNS production can stimulate persisters growth resumption, thereby resensitizing them to the action of bactericidal antibiotics and improve the effectiveness of treatment (73). Therefore, given that anti-infection therapy remains the core treatment for CAP, it is crucial to modulate oxidative stress to address the diverse bacterial responses to antibiotics.

Viral infections are associated with reduced GSH levels, which cause a cellular redox imbalance and activate a vicious cycle of reduced host defenses, leading to a severe imbalance in the intracellular microenvironment, and adjunctive antioxidant treatment may improve the outcomes of antiviral therapies (77). In fungal infections, an emerging view is that ROS production, regulation, and response are central axes of the host–pathogen interaction (78). Both the host and fungi produce ROS, use conserved mechanisms to detoxify ROS, and leverage ROS in their local environment to mediate defense mechanisms. In conclusion, oxidative stress is one of the important mechanisms in the development and progression of CAP, and the inflammatory response caused by lung lesions can exacerbate oxidative stress. On the basis of this situation, theoretically, exogenous supplementation with antioxidants is necessary along with the modulation of the immune and inflammatory responses. Moreover, an initial trial demonstrated that altered oxidative stress status is associated with the severity of CAP (5). Consequently, antioxidant therapy has been proposed as a supplementary treatment option.

## 5 Potential benefits and application value of modulating oxidative stress in older CAP patients

Global aging is already a reality today, putting enormous pressure on every country's healthcare system and representing an enormous challenge to global public health. The number of intensive care unit hospitalizations due to CAP have been rising steadily in recent years, especially in the older population, where CAP is a common cause of hospitalization, readmission, and death (79). A survey indicated that nearly 1 million older people in the U.S. are hospitalized for CAP each year, and nearly one-third of them die within 1 year; the burden of CAP in the older population is enormous (80). Despite geographic differences, *Streptococcus pneumoniae* remains a major pathogen for all ages worldwide (81, 82). And older adults are also more vulnerable to the growing epidemic caused by viruses (83). Compared with younger patients, older patients have an increased risk of infection with other pneumonia pathogens, and infections with influenza virus and antibiotic-resistant pathogens should also be considered. However, the incidence of so-called atypical pathogens is relatively low, and Mycoplasma infections are particularly rare (84). Early and appropriate anti-infective therapy remains a core part of the CAP treatment regimen. However, inappropriate antibiotic use is common in older adults (>65 years old), and older adults are particularly vulnerable to severe antibiotic-related adverse effects (85). Moreover, bactericidal antibiotics (quinolones, β-lactams, and aminoglycosides) have been found to cause ROS overproduction and mitochondrial dysfunction in mammalian cells, leading to oxidative damage to membrane lipids, proteins, and DNA (86).

Additionally, the aging process is accompanied by chronic inflammation and immune senescence (87). It has already been made clear that CAP is closely related to the immune response and inflammation, and the presence of an inadequate immune response together with an inflammatory flare-up can lead to a worse outcome (88). Therefore, older patients are at greater risk. On the basis of these findings, clinical intervention involving immunomodulation and suppression of the inflammatory response in older adults with CAP, such as the use of immunoglobulins and cortisol, would theoretically be beneficial but requires careful evaluation before use and has limited applicability (89, 90). Attention should also be paid to the fact that inappropriate treatment of patients hospitalized with CAP prolongs hospitalization and increases costs and mortality rates (82). In addition, with respect to older age groups, the fact that the nutritional status of older age groups, multiple age-related diseases, and chronic underlying conditions all have an impact on the onset and progression of CAP in older adults cannot be ignored (84). The positive effects of modulating oxidative stress in sepsis, *acute respiratory distress syndrome* (ARDS) and acute lung injury have been clinically demonstrated. It improves oxygenation rates and GSH levels and strengthens the immune response, and it reduces mechanical ventilation time, the length of stay in the intensive care unit, multiple organ dysfunctions, the length of stay in the hospital and mortality rates in acute lung injury/ARDS patients (84).

In conclusion, oxidative stress is involved in several aspects of CAP, including immunity and inflammation. Given the special risk factors and manifestations of CAP in older individuals and the existing studies, modulating oxidative stress may have the following advantages and application value (Table 1).

**Table 1:** Summary of pilot studies on vitamins C and E, NAC, CoQ10, and melatonin discussed in this article.

**Constituent**	**Study design**	**Description**	**Main results and finding**	**Experimental limitations**	**References**
Vitamin C	Clinical trial	Whether 2.0 grams/day of VitC prophylaxis versus placebo over 8 weeks reduces the incidence or severity of the common cold and other respiratory illnesses in 674 marine recruits.	Whole blood ascorbic acid levels were significantly higher in the VitC group 6 weeks after study initiation. No differences in cold incidence or duration were observed between groups, but cold symptoms were less severe in the VitC group. This study and the literature do not support using VitC prophylactically to prevent the common cold.	Study population and age limitations.	Pitt et al. (91)
	Clinical trial	Fifty-seven older adult patients admitted for acute respiratory infections (bronchitis and bronchopneumonia) received either 200 mg/day of VitC or placebo.	This modest oral dose significantly increased plasma and white cell VitC concentrations, even during acute respiratory infection. Patients receiving VitC supplementation showed significantly better clinical outcomes based on a symptom-based scoring system, especially those who were most severely ill at trial onset.	Enrolled patients with bronchitis may affect experimental results.	Hunt et al. (92)
	Cellular experiment (human and mice)	The study included 45 patients: 15 with normal status, 15 with non-severe CAP, and 15 with severe CAP. Lung tissues, blood specimens, and cultured murine alveolar macrophage cell lines were obtained from the patients and treated with lipopolysaccharide (LPS) and VitC. Various indicators of inflammation and oxidative stress were assessed.	An *in vitro* cellular assay confirmed that VitC reduced ROS and DNA damage in peripheral blood mononuclear cells and decreased TNF-α and IL-6 levels in whole blood cells from severe CAP patients. TNF-α, p38, and phosphorylated p38 levels were increased in LPS-stimulated macrophages. VitC reduced ROS levels, DNA damage, and the expression of TNF-α, p38, and phosphorylated p38 in LPS-stimulated macrophages *in vitro*.	Not mentioned.	Hunt et al. (93)
	Clinical trial	Ultra-high-performance liquid chromatography-tandem mass spectrometry (UPLC-MS/MS) was used to measure plasma VitC concentrations in 25 COVID-19 patients treated with VitC and 6 untreated, as well as in 60 healthy volunteers (51 for VitC measurement, 6 for blank plasma, and 3 for stability analyses).	COVID-19 patients had mean plasma VitC concentrations nearly five times lower than healthy volunteers. High-dose VitC treatment normalized and even exceeded healthy levels. Thus, 100 mg/kg/day VitC supplementation is essential for COVID-19 patients.	The trial was not a multicenter randomized controlled trial and recruited only non-smoking females to eliminate smoking effects on VitC concentrations.	Xing et al. (94)
	Animal study (mice)	Rats in the hepatic ischemia-reperfusion model received different doses of ascorbic acid (AA) before ischemia (30, 100, 300, and 1,000 mg/kg).	Hepatoprotective effects were observed only at low AA doses (30 or 100 mg/kg), whereas high doses (1,000 mg/kg) exacerbated ischemia/reperfusion-induced hepatic injury via lipid peroxidation. AA primarily acts as an antioxidant at low doses but exhibits pro-oxidant effects at high doses; thus, its use in pharmacological doses should be approached with caution.	Not mentioned.	Seo et al. (95)
	Clinical trial	The study included 60 patients (30 in each group) aged ≥18 years with moderate to severe COVID-19, hospitalized in the intensive care unit. The intervention group received intravenous VitC (1g q8h) for 4 days, while the placebo group received saline.	Although the intervention group had more severe cases, mortality was lower. High-dose intravenous VitC may reduce inflammation, improve oxygen support, and lower mortality in COVID-19 patients without adverse effects, suggesting its potential as a promising therapy for moderate to severe cases.	These results did not reach statistical significance due to the small sample size and moderate dose of intravenous VitC.	Kumar et al. (96)
	Clinical trial	Sixty patients with severe COVID-19 were randomized into control and intervention groups. The control group received lopinavir/ritonavir and hydroxychloroquine, while the intervention group received high-dose intravenous VitC (6 g/day) in addition to the same regimen.	On day 3, the intervention group showed greater improvements in mean body temperature and peripheral capillary oxygen saturation compared to the control group (*p* < 0.05), but had a significantly longer median hospital stay (*p* = 0.028). No significant differences were observed between groups in SpO_2_ levels at discharge, ICU length of stay, or mortality. No adverse effects were reported.	Limitations include small sample size and subjective evaluation indicators.	JamaliMoghadamSiahkali et al. (97)
Vitamin E	Clinical trial	Patients aged ≥65 years hospitalized for CAP were followed for up to 30 days from initial hospitalization for mortality. Those discharged alive within 30 days were followed for up to 90 days for re-hospitalization.	The study included 717 patients. VitE supplementation reduced the risk of re-hospitalization for any cause following discharge after pneumonia (P = 0.028). Neither influenza nor pneumococcal vaccines showed any protective effects on mortality or re-hospitalization.	Limitations included: (1) rare mortality and re-hospitalization events; (2) significant bias in assessing modifiable factors; (3) small sample size and patients lost to follow-up; (4) unconsidered impact of complications, overlapping infections, and adverse events.	Neupane et al. (98)
	Clinical trial	The study included 7,469 male smokers aged 50–69 years who had smoked since at least age 21. Participants received 50 mg/day of VitE for 5–8 years. The primary outcome was the incidence of hospital-treated CAP assessed at follow-up.	Among 2,216 participants who smoked 5–19 cigarettes/day and exercised, VitE supplementation reduced pneumonia incidence by 69% and prevented pneumonia in 12.9% of those taking VitE before age 74. Among 5,253 participants who smoked ≥20 cigarettes/day or did not exercise, VitE reduced pneumonia incidence by 14%. Overall, pneumonia incidence was 72% lower in patients using VitE, including those who smoked ≥20 cigarettes/day or did not exercise.	Limitations: The study only included older male smokers and did not involve non-smokers.	Hemilä (99)
	Clinical trial	The effect of VitE (50 mg/day) on the onset of pneumonia was examined in 133 male smokers aged 50–69 years, with the first occurrence of hospital-treated pneumonia assessed over a 3-year follow-up period.	VitE (50 mg/day) did not affect pneumonia risk in participants with physically demanding jobs but reduced the risk by 50% in those engaging in moderate or heavy exercise during leisure time	No definitive practical conclusions can be drawn from this subgroup finding, and further studies are warranted among individuals engaging in moderate or heavy exercise.	Milisav et al. (100)
	Animal study (mice)	To establish an aging model, 6-week-old female mice were continuously administered d-galactose (d-gal). The VitE group received intragastric α-Tocopherol (50 mg/100 mL/kg) diluted in highly refined, low-acidity olive oil, along with daily intraperitoneal d-gal for 4 weeks. Levels of advanced glycation end products (AGEs) in the blood, T cell phenotypes in the thymus and spleen, and ROS levels in spleen-derived leukocytes and T cells were measured as indices of aging and immunosenescence.	After 6 weeks of d-gal administration, intracellular ROS levels in the mouse spleen and serum AGEs were significantly higher. Thymic function was reduced, and peripheral T cell subsets redistributed. VitE treatment significantly reduced ROS levels (P < 0.05) and decreased serum AGEs, while increasing the naïve to effector-memory T cell ratio in aging mice.	Limitations of this study include not considering the effects of gender, age, and treatment duration on D-gal-induced oxidative stress. Additionally, the research did not provide further evidence of an aging phenotype beyond serum AGE levels.	Lee et al. (101)
	Cellular experiment (human skeletal muscle myoblasts)	Human skeletal muscle myoblasts in the replicative senescent state were treated with tocotrienol-rich fraction (TRF), an ubtype of VitE.	Replicative senescence increased ROS generation and lipid peroxidation in myoblasts. TRF treatment significantly reduced ROS production and lipid peroxidation. It also modulated the gene expression of SOD, CAT, and GPX, increasing superoxide dismutase and catalase activity while reducing glutathione peroxidase activity. TRF improved antioxidant defenses and mitigated oxidative stress associated with replicative senescence in myoblasts.	Not mentioned.	Khor et al. (102)
Vitamin E	Clinical trial	Among 817 eligible older adults categorized into frail and non-frail groups, plasma VitE levels were measured. Confounders assessed included lower extremity muscle strength, cognitive function, diseases, and factors related to VitE metabolism.	In a logistic model adjusted for multiple confounders, participants in the highest VitE tertile were less likely to be frail compared to those in the lowest tertile. Oxidative stress may contribute to frailty development. VitE: Low circulating levels of a key component of the human antioxidant system are associated with frailty. VitE may be associated with frailty through an unknown mechanism independent of its antioxidant properties.	Important factors were not considered, and confounder measures lacked precision, potentially leaving residual confounding. Other antioxidant pathways were not assessed. The cross-sectional study design precludes ruling out reverse causality.	Ble et al. (103)
Vitamin C and Vitamin E	Animal study (mice)	In an oleic acid (OA)-induced ARDS rat model of acute lung injury, control groups received no treatment, while experimental groups (*n* = 6 each) received VitE (100 mg/kg) or VitC (100 mg/kg).	OA increased lung MDA levels and serum TNF-α and IL-1β levels, while VitE and VitC significantly reduced cytokine levels. OA also decreased lung SOD, CAT, and GSH levels, which were significantly restored by vitamins. Histopathological evidence showed reduced neutrophil infiltration in VitE and VitC groups compared to the OA group. Additionally, these groups exhibited less endothelial and tissue damage, as indicated by decreased bronchial and alveolar septum thickening, vascular hyperemia, and polynuclear cell infiltration.	Not mentioned.	Erol et al. (104)
	Animal study (mice)	The dorsiflexors of the left limbs in young and aged male rats were subjected to repetitive loading exercise. Seven animals from each age group were randomly assigned to either a diet supplemented with VitE (DL-alpha tocopheryl acetate; 30,000 mg/kg) and VitC (L-ascorbic acid; 2% by weight) or normal non-supplemented (NS) rat chow containing 126 mg/kg VitE and no VitC. All animals had free access to rat chow and water. Biomarkers of oxidative stress were measured in the tibialis anterior muscles.	Positive work increased only in aged animals supplemented with VitE and C. Markers of oxidative stress increased in the tibialis anterior muscles of aged and young adult animals subjected to repetitive loading, but VitE and C supplements attenuated this increase. CuZnSOD and CAT activities increased with supplementation in both young adult and aged animals. These data suggest that antioxidant supplementation improves indices of oxidative stress associated with repetitive loading exercise and aging and enhances the positive work output of muscles in aged rodents.	VitE and C levels were not measured in the skeletal muscles of experimental animals.	Ryan et al. (105)
	Clinical trial	Forty male patients with type 2 diabetes aged 40–60 years, receiving antidiabetic therapy with 500 mg of metformin tablets twice daily (BID). The patients were randomly divided into four groups: Control group: 500 mg of metformin + placebo BID; VitC group: 500 mg of metformin + 500 mg of VitC BID; VitE group: 500 mg of metformin + 400 mg of VitE BID; Vitamins C + E group: 500 mg of metformin + 500 mg of VitC + 400 mg of VitE BID. All groups received the treatments for 90 consecutive days.	VitC and/or E improved fasting blood sugar (FBS), HbA1c, lipid profile, insulin, homeostasis model assessment of insulin resistance (HOMA-IR), reduced glutathione (GSH), and Quantitative Insulin Sensitivity Check Index (QUICKI) compared with the diabetic group receiving placebo. This study provides additional evidence that antioxidant vitamin supplementation in type 2 diabetes mellitus (T2DM) can improve clinical outcomes, attenuate or prevent diabetic pathogenesis and complications, and address the imbalance between declining endogenous antioxidants and increasing ROS production, which is exacerbated by poor glycemic control and contributes to oxidant-mediated damage in diabetes.	The experimental sample size was small and included only males.	El-Aal et al. (106)
NAC	Cellular experiment	Intervention with the antiviral drugs raltegravir (Rem), nelfinavir (Nel), and NAC in SARS-CoV-2-infected Vero E6 cells.	SARS-CoV-2 infection impairs cellular glutathione metabolism. NAC and the antiviral drug Nel can prevent this defect *in vitro*.	A limitation of this study is the use of Vero E6 cells instead of a cell model that more closely represents the lung cell targets of SARS-CoV-2.	Bartolini et al. (107)
NAC	Clinical research	The 217 patients hospitalized with COVID-19 in the intervention group received 1,500 mg of NAC intravenously daily, while the 245 patients hospitalized with COVID-19 in the control group did not receive NAC. Outcomes were assessed after 10 consecutive days of treatment.	Compared with the control group, the NAC group showed significant improvements in median room air SpO_2_, median SpO_2_/FiO_2_ ratio, length of hospitalization, and incidence of cough, dyspnea, and loss of appetite (P < 0.05). NAC is a safe, tolerable, available, and affordable drug that appears to be a promising adjuvant therapeutic agent for COVID-19.	This study was unicentric, and the efficacy of NAC in combination with remdesivir and steroids was not evaluated.	Afaghi et al. (108)
	Animal study (mice) and cellular experiment (bovine embryo tracheal cells)	Bovine embryonic tracheal cells (EBTr) and a mouse lung injury model, both induced with lipopolysaccharide (LPS), were treated differentially with NAC.	NAC pretreatment attenuated LPS-induced inflammation in both EBTr cells and mouse models. Moreover, LPS suppressed the expression of oxidative-related factors in EBTr cells while promoting the gene expression and secretion of inflammatory cytokines. inflammatory cytokines and decreased their mRNA levels, maintaining stable levels of antioxidative gene expression. Conversely, NAC pretreatment alleviated the secretion of inflammatory cytokines, decreased their mRNA levels, and maintained stable levels of antioxidative gene expression. *In vivo*, NAC mitigated LPS-induced inflammatory responses and lung injury in ALI mice. Compared with the LPS group, the NAC group exhibited significant decreases in relative protein concentration, total cell count, and percentage of neutrophils in BALF, as well as in the secretion levels of IL-6, IL-8, TNF-α, and IL-1β, MPO activity, lung injury score, and expression levels of inflammatory-related genes. NAC also ameliorated LPS-induced changes in mRNA levels of antioxidant genes. In conclusion, the findings suggest that NAC modulates both inflammatory and oxidative responses, thereby alleviating LPS-induced inflammation in EBTr cells and lung injury in mice.	Some results observed in EBTr cells were not reproduced in the LPS-ALI mouse model.	Chen et al. (109)
	Clinical trial	COVID-19 patients receiving high-dose oral NAC (600 mg every 8 h) were compared with those not receiving NAC.	The NAC group (*n* = 2,071) had a higher overall baseline risk but showed better performance in reducing mortality. This result remained significant in multivariate analyses adjusted for baseline characteristics and concomitant corticosteroid use. However, no significant differences were observed in mean length of hospitalization, admission to the intensive care unit, or use of invasive mechanical ventilation between the NAC and non-NAC groups. High-dose oral NAC treatment in admitted COVID-19 patients was associated with significantly lower mortality, despite these patients being older, more frequently male, and having more comorbidities. NAC has a favorable safety profile.	The study was observational and lacked complete patient information; thus, a controlled clinical trial is required.	Izquierdo et al. (110)
	Clinical research	In COVID-19 patients admitted to the ICU, 72 patients who received NAC for 3 days were compared with 68 patients who did not receive NAC.	NAC-treated patients exhibited higher PaO_2_/FiO_2_ ratios after 3 days in the ICU compared with the control group (*p* < 0.05). The administration of NAC improved the clinical and analytical responses in critically ill COVID-19 patients compared with the control group. NAC prevented the decrease in glutathione concentrations.	Limitations included the absence of data on patients with mild symptoms, the single-center design of the trial, and the lack of sensitivity in the GSH measurement method.	Gamarra-Morales et al. (111)
Coenzyme Q10	Cellular experiment (human lung, mice lung)	This study examines the effects of *P. aeruginosa* on lipid peroxidation in human and mouse lungs, as well as cell death induced by *P. aeruginosa* in human airway epithelial cells.	Lipid peroxidation was detected in human cystic fibrosis (CF) lungs and correlated with bacterial infection. Incubation of CF bronchial epithelial cells with *P. aeruginosa* induced an increase in ROS, leading to lipid peroxidation and cell death. Antioxidants, such as CoQ10, inhibited *P. aeruginosa*-induced cell death. Antioxidants are proposed as a treatment for pneumonia caused by *P. aeruginosa* infection.	Not mentioned.	Ousingsaw et al. (112)
	Clinical trial	Hospitalized older adults with CAP (diagnosed using defined clinical and radiological criteria) were randomized to receive oral CoQ10 (200 mg/d) or placebo for 14 days, in addition to antibiotics.	A total of 141 patients were included (70 in the CoQ10 group and 71 in the placebo group). Patients receiving CoQ10 recovered faster (*P* = 0.0206) and had a shorter hospital stay (*P* = 0.0144) compared with the placebo group than in the placebo group (*P* = 0.0440). The rate of treatment failure was lower in the CoQ10 group than in the placebo group (*P* = 0.0440). CoQ10 administration has no serious side effects and improves outcomes in hospitalized older adults with CAP; therefore, we recommend it as an adjunctive treatment in this population.	Limitations include the lack of assessment for CoQ10 deficiency, failure to identify bacterial pathogens, and the unexplored effect of specific bacterial pathogens on CoQ10 efficacy. Additionally, the use of clinical cure as the primary outcome is subjective and may introduce bias.	Farazi et al. (113)
Melatonin	Cellular experiment	A mouse model of Klebsiella pneumoniae infection was established. Control mice were left untreated, while experimental mice were treated with 2 mg/kg melatonin via oral gavage for 6 consecutive days. Corresponding tissues and cells were collected after treatment.	Melatonin treatment significantly upregulated telomerase activity in macrophages, which was associated with reduced ROS levels and enhanced cellular energy production, indicating improved mitochondrial function. Moreover, melatonin treatment suppressed NLRP3 inflammasome activation, resulting in reduced IL-1β secretion. These findings suggest a potential therapeutic role for melatonin in pneumonia treatment.	The experiments were conducted *in vitro* using macrophage cell lines, which may not fully reflect the complexity of the *in vivo* environment. Insights into potential mechanisms were provided, but further investigation is needed.	Jiang et al. (114)
	Clinical research	Seventy-four hospitalized patients with mild to moderate COVID-19 were divided into test and control groups (37 patients each). Both groups received standard treatment, while the test group additionally received melatonin (3 mg, three times daily) for 14 days.	A total of 24 patients in the intervention group and 20 in the control group completed the treatment. Compared with the control group, the intervention group showed significant improvements in clinical symptoms (cough, dyspnea, malaise), CRP levels, lung involvement, mean time to discharge, and return to baseline health (p < 0.05). There were no deaths or adverse events in either group.	The main limitations of this trial were the small sample size and short-term follow-up, which may introduce bias.	Farnoosh et al. (115)
	Clinical research	Patients admitted to the ICU with severe COVID-19 were divided into two groups: 109 in the melatonin group and 117 in the control group. Both groups received conventional therapy, while the melatonin group additionally received oral melatonin (5 mg, twice daily) for 7 days.	Compared with the control group, patients in the melatonin group showed significant improvements in mortality, rate of mechanical ventilation, median days to discharge, and time to ≥2-point improvement in clinical status (P < 0.05). Dizziness was the most common side effect in the melatonin group. Melatonin significantly improved clinical status and was well-tolerated in patients with severe COVID-19 pneumonia.	Further trials with larger sample sizes, diverse populations, and high-quality designs are needed to confirm these preliminary findings.	Ameri et al. (116)
	Clinical research	A total of 40 hospitalized patients with COVID-19 completed the study, with 20 patients in each of the intervention and control groups. Both groups received standard treatment, while the intervention group additionally received oral melatonin (9 mg/day) for 14 days. Blood samples were collected from patients at the beginning and end of the treatment period.	Adjuvant therapy with melatonin controlled and reduced inflammatory cytokines in COVID-19 patients. Melatonin also controlled and modulated the dysregulated genes involved in Th1- and Th2-mediated humoral and cellular immunity. This study demonstrated for the first time that melatonin can serve as an anti-inflammatory adjuvant by regulating the expression of Th1 and Th2 regulatory genes to reduce and control inflammatory cytokines in COVID-19 patients.	Limitations of the study included restricting participants to patients admitted at the start of the outbreak, excluding patients with severe symptoms, not assessing the effect of melatonin dosage, and not examining CD4+, CD8+ T cells, or NK cells.	Hosseini et al. (117)
Melatonin	Clinical research	Seventeen COVID-19 patients receiving melatonin treatment (5 mg, q12h, for 5 days) were compared with 20 healthy subjects.	The activities of antioxidant enzymes were decreased after melatonin treatment (*P* < 0.05). Melatonin treatment enhanced antioxidant enzyme activity, contributing to increased total antioxidant capacity and restoration of redox homeostasis. Thus, melatonin restores redox homeostasis altered in COVID-19 patients and can serve as an adjuvant therapy for SARS-CoV-2 infection.	Limitations of the study include the small sample size of COVID-19 patients, insufficient duration of antioxidant therapy due to budget constraints, and the absence of postmortem samples treated with melatonin using the immune colloidal gold technique to demonstrate prevention of mitochondrial sequestration.	Soto et al. (118)
Melatonin	Animal study (mice)	Middle-aged mice were divided into three groups (six mice per group). The control group was untreated, while the experimental groups received melatonin (MEL) at 5 mg/kg/day and 10 mg/kg/day by gavage for 2 months, respectively.	Melatonin (MEL) alleviated histological damage and increased the cross-sectional area of muscle fibers in gastrocnemius (GA) tissues of middle-aged mice. Furthermore, MEL treatment increased the percentage and size of normal mitochondria and mtDNA copy number while reducing MDA and ROS levels in GA tissues of middle-aged mice.	Not mentioned	Fang et al. (119)

Note that the findings presented in this table are not exhaustive, and some experiments do not mention study limitations.

### 5.1 Preventive value of CAP in older adults

Regardless of what disease you are facing, the first tool to consider should be prevention. Vaccines, as the only effective preventive medical treatment, can reduce the risk and burden of contracting diseases such as influenza and pneumococcal pneumonia (120). An analysis of national pneumococcal vaccination data in Korea revealed that previous pneumococcal vaccination in the older population effectively improved hospitalization rates and 30-day mortality among patients hospitalized for pneumonia (121). However, the outcomes of pneumococcal vaccine use in older adults remain controversial. A retrospective study in Japan concluded that the effectiveness of pneumococcal vaccines was significant in people aged 65–85 years but not in the older population (122). However, vaccine coverage is not satisfactory, and increasing vaccination rates remains a difficult challenge (123). Moreover, vaccines do not cover all pathogens and take a certain amount of time to develop. When faced with an outbreak of a worldwide disease such as COVID-19, the use of a vaccine can be caught off guard (124).

Oxidative stress is one of the important mechanisms in the development of CAP, and maintaining the balance of the oxidative state in the body may be able to produce a preventive effect through a different mode of action than vaccines do. Moreover, in the older population, there is a low-grade chronic inflammatory state formed by senescent cells stimulating the secretion of proinflammatory cytokines. Individuals with low-grade chronic inflammation present a dysregulated innate immune system, resulting in an increased risk of infection (125). Meanwhile, the subsequent inflammatory cascade further increases extracellular ROS concentrations and oxidative stress (126). From this perspective, modulating oxidative stress can also play a role in preventing the onset and controlling the progression of CAP in older individuals by controlling chronic inflammation and reducing the accumulation of ROS.

For example, vitamins C and E are the most common antioxidants. VitE is a fat-soluble antibiotic whose main role is to prevent lipid peroxidation, which minimizes the formation of secondary radicals. Alpha-tocopherol is the most potent antioxidant in the VitE composition and can react quickly with free radicals to generate relatively stable tocopheroxyl radicals, thus improving oxidative stress (33). An earlier prospective cohort study also confirmed that VitE supplements prevented rehospitalization in patients (>65 years old) who were first hospitalized for CAP (98). In another follow-up study, VitE (50 mg/d) was administered for 5–8 years to older male smokers (50–69 years old), which revealed that VitE administration reduced the incidence of pneumonia in older men in different subgroups (99). However, some studies have noted that the effect of VitE on the incidence of pneumonia does not seem to be completely uniform (127–129). An early clinical trial found that VitE (50 mg/day) did not affect pneumonia risk in participants with physically demanding jobs but reduced the risk by 50% in those engaging in moderate or heavy exercise during leisure time (129). Another study found that VitE reduced the risk of pneumonia by 69% in participants who smoked the least and exercised in their leisure time but increased the risk of pneumonia by 68% in participants who smoked the most and did not exercise (130). However, the study focused only on Finnish male smokers aged 50–69 years, and long-term high-frequency smoking is more directly and detrimentally associated with the development of pneumonia.

VitC can directly quench free radicals in the aqueous layer and also cooperate with VitE to regenerate α-tocopherol from α-tocopherol radicals in membranes and lipoproteins and increase GSH levels in the cell; thus, it plays an important role in protein thiol group protection against oxidation (33). One study reported that VitC can effectively kill bacteria, mycobacteria, HIV, and HCV because it can generate free radicals and H_2_O_2_ (131). As such, VitC plays a number of important roles in reducing oxidative stress caused by infection, balancing the immune system, and killing microorganisms by generating free radicals. However, the role of VitC in preventing pneumonia is not very certain and is still only a reliable speculation that lacks confirmation from more reliable and relevant experimental studies. In an earlier study, 674 Marine Corps recruits received 2 g/day of VitC for 8 weeks. The study found that VitC did not significantly alter the incidence or duration of colds, but it did reduce the severity of cold symptoms in the treatment group (91). A recent meta-analysis emphasized that the current evidence is insufficient to affirm the efficacy of VitC supplements in preventing pneumonia because of the small number of trials and the very low quality of the available results (132). However, a recent review confirmed the preventive role of VitC in viral pneumonia through a different lens. Owing to pneumonia, viral infections can reduce the level of ascorbic acid because they create oxygen (O) and nitrogen (N) species (133). Therefore, VitC can prevent pneumonia. In addition, an analysis of nationally representative data from more than 34,000 participants revealed that low serum levels of antioxidant vitamins were associated with increased respiratory morbidity and/or mortality in U.S. adults (134). These results underscore the importance of antioxidant vitamins in respiratory health.

There are many other important antioxidants, including the previously mentioned GSH, CoQ10, which is present in all membranes of the cell, and melatonin. The powerful antioxidant effects of GSH are not repeated. Increasing GSH levels for the prevention and inhibition of COVID-19 are also widely recognized (135). *N-Acetyl-L-cysteine* (NAC) is a precursor of reduced GSH. NAC has better oral and topical bioavailability than GSH, and has an excellent safety profile and is also a mucolytic agent (136). NAC is able to act as a potent antioxidant scavenger of free radicals directly but also reduces downregulated inflammation and lowers the level of oxidative stress through stimulation of Nrf2 and inhibition of the NFκB pathway (137). NAC, a pleiotropic drug, has been proposed for the treatment and/or prevention of a variety of diseases involving GSH depletion and altered redox status. For example, *severe acute respiratory syndrome coronavirus 2* (SARS-CoV-2) infection impairs the metabolism of cellular GSH, and NAC can prevent such defects *in vitro* (107).

CoQ10 has potent antioxidant effects on the mitochondrial membrane, as well as other membranes in the cell and in the plasma and cytoplasm. Its most important and relevant role is the ability to switch between the redox forms (ubiquinone, semiubiquinone, and ubiquinol) and maintain a balance between the redox forms outside the mitochondria through enzymatic action (138). A molecular review revealed that CoQ10, which reduces important inflammatory cytokines and prevents organ damage due to massive oxidative stress, has been tested for the prevention of a wide range of diseases, especially those with an inflammatory pathogenesis. CoQ10 supplementation could prevent COVID-19-induced morbidities and potentially has a protective role against the deleterious consequences of the disease (139).

Melatonin is secreted mainly by the pineal gland of the brain, and its secretion is extremely high in infants and adolescents and much lower in older individuals. Melatonin scavenges free radicals directly and also stimulates a variety of antioxidant enzymes, including SOD, GPX, glutathione reductase, and CAT, inhibits pro-oxidant enzymes and increases intracellular GSH levels. There is also evidence that melatonin stabilizes cell membranes, which may help fight oxidative damage (33). A review summarizes the potent bacteriostatic effects of melatonin, such as resistance to pathogenic bacterial infections *in vivo* via various pathways, including NF-κB and ROS; antimicrobial activity against classical gram-negative and gram-positive bacteria, even members of other bacterial groups such as *Mycobacterium tuberculosis*; and antiviral effects (140). Although there is no experimental confirmation of the effectiveness of the antioxidant effects of melatonin in the prevention of CAP. However, melatonin has demonstrated satisfactory results in terms of being a possible prophylactic measure for COVID-19 infection (141). This may be related to its ability to reduce mitochondrial oxidative stress in posterior lung cells after COVID-19 infection while conferring a general antioxidant effect (142). A retrospective cross-sectional study analyzed data from a closed population of 110 adult patients treated with melatonin for a variety of sleep disorders until the onset of the COVID-19 pandemic (143). The patients in general were mostly older adults treated with a mean dose of melatonin >40 mg/day for various sleep disorders, mainly for complaints of insomnia, for more than 12 months. COVID-19 infection was recorded in 15 patients (13.5%) requiring hospitalization, 5 of whom were infected, only one with severe pneumonia and no deaths due to COVID-19. These results are consistent with a possible preventive effect of melatonin in the context of the COVID-19 pandemic.

The previously mentioned representative antioxidants, while playing an antioxidant role, are also nutrients needed by humans and can indirectly improve the state of malnutrition in the older population, further affecting immune function and playing a preventive role (65). Certainly, the modulation of oxidative stress works in an indirect, slow, and gentle manner as opposed to the direct stimulation of the immune response by vaccines. In addition, some vaccines produce effects by exerting antioxidative stress effects (144). Theoretically, vaccines are synergistic with antioxidative stress, and the significance and value of modulating oxidative stress in preventing CAP in older individuals may be better realized on the basis of the low-grade chronic inflammatory state and the chronic accumulation of ROS in older individuals. However, because we have only just experienced COVID-19, there will be a relatively greater number and depth of articles on prophylactic research related to viral CAPs such as pneumonia with COVID-19. This enthusiasm should be maintained for research on other pathogens, including bacteria and fungi. The more we understand about biology, the better we are equipped to meet sudden public health challenges such as the COVID-19 pandemic (145).

### 5.2 Reducing lung inflammation

Inflammation and oxidation are apparently interrelated processes, as excessive or uncontrolled free radical production induces an inflammatory response, while free radicals are inflammatory effectors. In fact, both oxidation and inflammation occur when the immune system responds to pathogen invasion (146). An analysis of endogenous oxidative damage markers and their associations with pulmonary involvement severity in patients with SARS-CoV-2 pneumonia indicated that SARS-CoV-2 pneumonia is significantly associated with increased endogenous oxidative damage. Oxidative damage seems to be associated with the severity of pulmonary involvement (147). Many compounds with antioxidant effects, such as those mentioned above, that protect lung tissue from CAP by attenuating oxidative stress-induced damage and inhibiting inflammatory responses have been identified.

According to the results of an animal study in rats, the oral administration of antioxidant vitamins such as VitE and VitC may help inhibit the development of ARDS (104). An early randomized, double-blind trial of 57 older adult patients admitted to the hospital for acute respiratory infections (bronchitis and bronchopneumonia) using a clinical scoring system based on major symptoms of the respiratory condition revealed that VitC-supplemented patients fared significantly better than placebo-supplemented patients did (92). An *in vitro* assay confirmed that VitC reduced ROS and DNA damage in peripheral blood mononuclear cells and decreased TNF-α and IL-6 levels in whole blood cells from patients with severe CAP (93). A research has found that COVID-19 patients have an average plasma VitC concentration nearly five times lower than that of healthy volunteers. Therefore, it is recommended that COVID-19 patients receive VitC supplementation at a dose of 100 mg/kg per day (94). NAC is a safe, tolerable, available and affordable drug which seems like a promising adjuvant therapeutic agent for COVID-19 (108). In animal experiments, NAC was found to attenuate lipopolysaccharide-induced inflammation in bovine embryonic tracheal cells and lung injury in mice (109). In the clinical trial, the group of COVID-19 patients treated with NAC had a greater risk at baseline, such as being older (average age over 70) and suffering from more comorbidities such as hypertension, diabetes, and COPD. However, patients who received NAC had a significantly lower mortality rate than did COVID-19 patients who did not receive NAC, which was attributed to the antioxidative stress effect of NAC (110). Treatment with NAC in critically ill patients with COVID-19 (mean age >60 years in both experimental and control patients) improved patients' PaO_2_/FiO_2_ indices, and it was hypothesized that NAC prevented the reduction in GSH and thus affected clinical outcomes (111). In mouse experiments, CoQ10 significantly inhibited *Pseudomonas aeruginosa*-induced cell death; thus, antioxidants such as CoQ10 have been proposed for the treatment of pneumonia caused by *Pseudomonas aeruginosa* infection (112). CoQ10 treatment in older adults with CAP accelerated symptom resolution, shortened the duration of antibiotic therapy, and also performed well in the subgroup of patients with severe pneumonia (113). In cellular experiments, melatonin can upregulate telomerase activity in macrophages by decreasing ROS levels, increasing cellular energy production, and inhibiting NLRP3 inflammatory vesicle activation in macrophages, suggesting that melatonin has therapeutic potential for pneumonia (114). In clinical trials, adjunctive treatment with melatonin has demonstrated efficacy in patients with mild, moderate, and severe COVID-19 (115, 116). Melatonin can be used as a medicinal adjuvant with an anti-inflammatory mechanism to reduce and control inflammatory cytokines by regulating the expression of *T helper 1* (Th1) and Th2 regulatory genes in patients with COVID-19 (117). The results of a recent clinical trial revealed that melatonin restores redox homeostasis, which is altered in COVID-19 patients and can be used as an adjuvant therapy for SARS-CoV-2 infection (118).

Indeed, modulating oxidative stress plays a role primarily in controlling the inflammatory response component of CAP. A range of lung damage caused by inflammation and oxidative stress can be reduced, which can improve clinical symptoms in older patients. Previous studies have theoretically and experimentally demonstrated the positive therapeutic effects of modulating oxidation in viral CAPs, such as COVID-19, but there is a relative lack of more relevant clinical studies to further clarify the clinical efficacy in CAP infection caused by other pathogens.

### 5.3 Immunosenescence-related benefits

**Figure 4:**
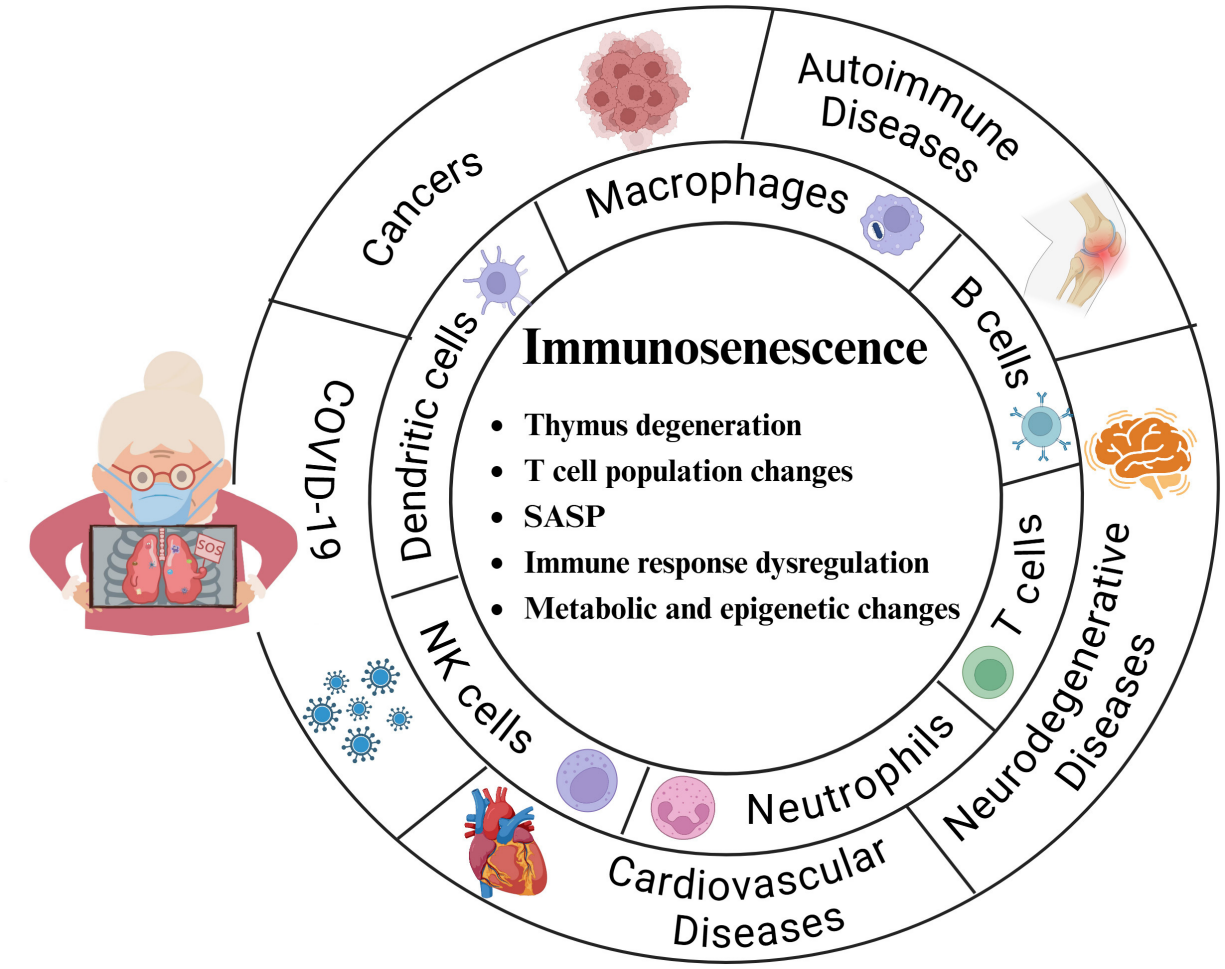
Main features of immunosenescence and the major cells and diseases affected. **Thymic degeneration:** The thymus gland is essential for T-cell development, but it gradually shrinks and degenerates with aging, leading to a decrease in the production of new T cells. **T-cell population changes:** An increase in memory T cells and a decrease in initial T cells weaken the ability to respond to new antigens. **Senescence-associated secretory phenotype (SASP):** This phenotype promotes cellular senescence, including immune cells, and promotes the secretion of proinflammatory cytokines, chemokines, and proteases that lead to chronic inflammation (inflammatory aging). **Immune response dysregulation:** Decreased immune surveillance, poor response to vaccines, increased susceptibility to infections, and increased incidence of autoimmune diseases and cancers. **Metabolic and epigenetic changes:** Alterations in metabolic and epigenetic pathways significantly affect immune system aging and T-cell senescence.

With respect to older adults, the topic of aging, especially immune aging, cannot be avoided. The immune system is widely believed to undergo quantitative and qualitative changes during the process of aging (148). This age-related decline in immune function, called immune senescence, leads to alterations in the cytokine microenvironment as well as impaired innate and adaptive immunity and is closely related to oxidative stress (149). According to existing studies, immune senescence is characterized mainly by major features, such as thymic degeneration, changes in T-cell populations, senescent secretory phenotypes, dysregulation of immune responses, and metabolic and epigenetic changes, which affect cardiovascular diseases, neurodegenerative diseases, COVID-19, autoimmune diseases, and cancer[(150–154), Figure 4].

In general, all immune cells are affected by aging (155). Compromised immune cells lose their ability to modulate their own redox and inflammatory homeostasis, which is characterized by mononuclear immune cells infiltrating different tissues and producing excessive amounts of ROS and proinflammatory mediators (156, 157). High levels of ROS in cells are considered the driving force behind the deleterious effects of the aging process (153). This may lead to impaired phagocytosis of macrophages, decreased production of lymphocyte factors and antibodies, dysregulation of inflammatory processes, and an impaired immune response (87). If these affected immune cells are not well modulated, they further increase oxidative and inflammatory stress, creating a perpetual cycle that increases the rate of aging (158). These immune senescence changes further reduce the ability of the lungs to respond to infection and repair damage, increasing vulnerability to respiratory disease and slowing recovery from illness (159).

In addition, one of the hallmarks of immune senescence is “inflammation,” which refers to an age-related systemic state of sterile, chronic and low-grade inflammation characterized by the upregulation of blood markers of inflammation and is considered to be a consequence of increasing chronological age, as well as a hallmark of biological senescence, multimorbidity and mortality risk (87, 153). And oxidative stress plays an important role in the development and maintenance of low-grade inflammation in age-related diseases. The ROS generated by oxidative stress activate the NF-κB signaling pathway, which drives the expression of many proinflammatory cytokine genes as transcription factors, leading to chronic sterile inflammation and significantly affecting the aging process (160). In fact, unstimulated neutrophils and lymphocytes isolated from older adults patients accumulate greater amounts of ROS, present decreased SOD activity, and are less resistant to cell death compared to those cells obtained from young individuals (161). In contrast, the feedback loop between ROS and NF-κB exacerbates oxidative damage and inflammation, leading to immune cell senescence and a decrease in tissue function. Together, oxidative stress and immune senescence form a vicious cycle in which impaired immune function increases susceptibility to oxidative damage, and oxidative stress further weakens the immune response (162).

In conclusion, the relationships among immune senescence, chronic inflammation and oxidative stress are intertwined and complex. While the links have not been fully elucidated, reducing systemic oxidative stress likely reduces the risk of stress-induced cellular senescence and associated inflammation (87). Notably, immune senescence is particularly detrimental to the respiratory system of older adults, who are easy targets for exogenous infections (162). Furthermore, due to immune senescence, older patients have an inadequate inflammatory response to infection, which may lead to an underestimation of the severity of pneumonia (163).

Some existing studies have also confirmed the effectiveness of counteracting immune senescence from the perspective of modulating oxidative stress. For example, flavonoids, which are natural antioxidants present in various foods, can act as antioxidants through signaling pathways such as the NF-κB pathway (164). Quercetin, a type of flavonoid, has been widely reported for its antioxidant activity (165). In an *in vitro* cellular assay, aged peripheral blood mononuclear cells treated with quercetin presented a strong proliferative response comparable to that of young cells and increased GSH antioxidant defense levels, significantly reducing the expression of immune senescence markers (166). Another botanical bioflavonoid composed of *Scutellaria baicalensis* and *Acacia catechu* improved innate and adaptive immunity through antioxidant effects in a rat model of immune senescence and has great potential for the treatment of respiratory disorders due to immune stress and aging (162). In immunosenescent mice, although not showing any statistical difference, the administration of VitE increased the ratio of naïve to effector-memory T cells, suggesting that VitE may be effective in restoring oxidative stress-induced immunosenescence (101). There are also studies confirming that melatonin supplementation can prevent or delay the deterioration of immune system function that accompanies aging and may restore it to a “youthful” state (149).

Indeed, chronic oxidative stress is only one of the many factors that contributes to immune senescence in the older population. Basic experiments have demonstrated that modulating stress can improve or even reverse immune senescence. However, this is destined to be a long process, and long-term clinical studies are needed to fully substantiate the value of antioxidant therapy.

### 5.4 Improvements in sarcopenia and frailty

**Figure 5:**
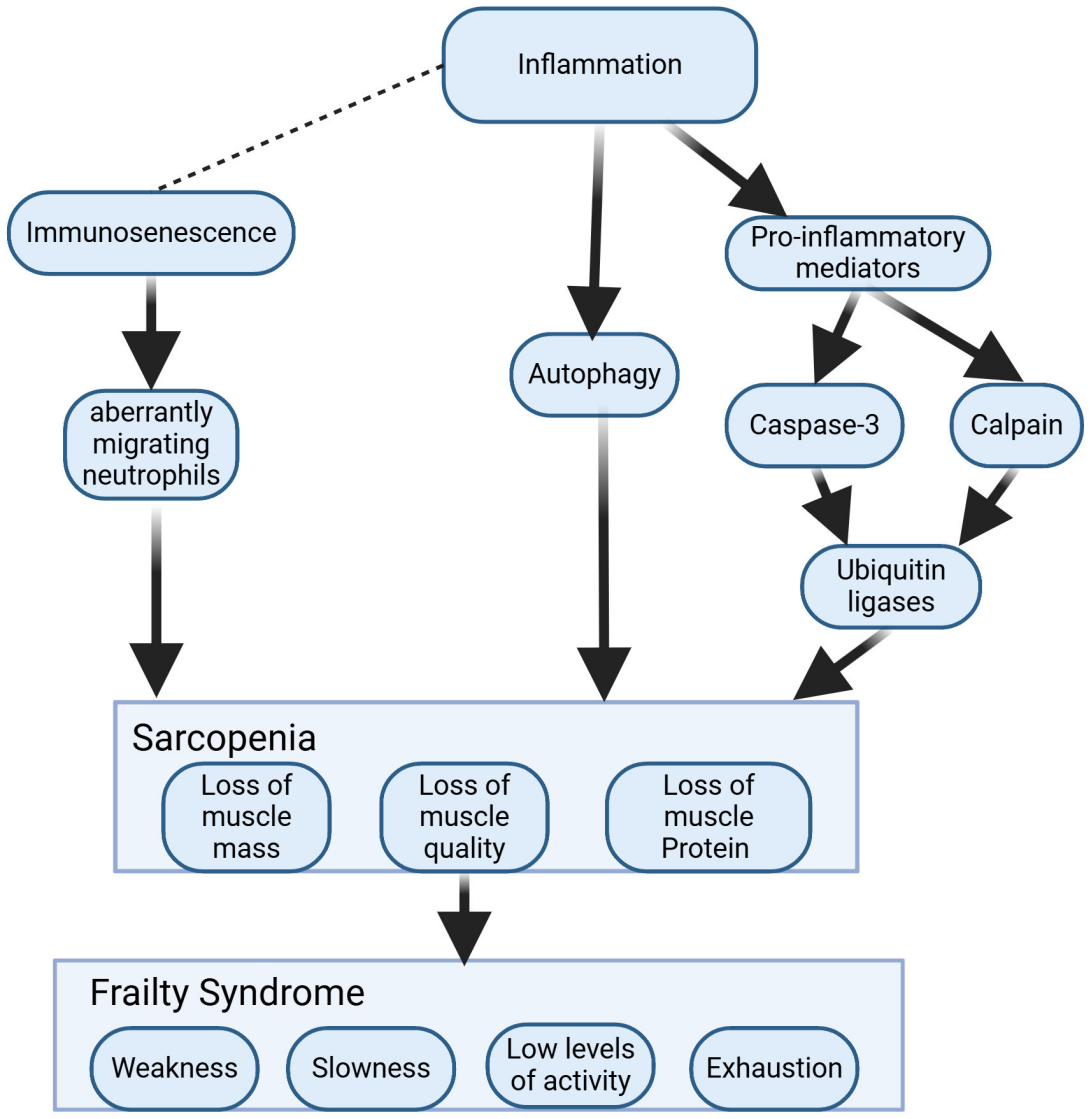
Mechanistic models of sarcopenia and frailty induced by inflammation and immunosenescence. Inflammation induces the production of proinflammatory cytokines and activates scalpains and caspase-3 to cleave myofibrillar proteins. The ubiquitin–proteasome system degrades cleaved proteins. Autophagy is another pathway of muscle atrophy caused by inflammation. Chronic low-grade inflammation causes immunosenescence, leading to aberrantly migrating neutrophils that cause secondary damage to healthy muscle. Sarcopenia can develop further into frailty if it is not promptly corrected.

In the case of older adults with CAP, to seek effective treatment strategies, it is necessary to consider not only pathogen infection but also functional decline. Cough is the most common and primary manifestation of CAP and is important for clearing secretions from the airways. The strength of the cough is regulated by the respiratory muscles, and a weakened cough reflex can lead to the development of pneumonia, especially aspiration pneumonia, in older individuals (167). Therefore, respiratory muscle weakness has also been shown to be a risk factor for the development of pneumonia and for the recurrence of pneumonia in older individuals (168). In addition, the role of the swallowing reflex is also important. It is another protective airway reflex, such as coughing, and its strength is related to the swallowing muscles (169). Both impaired reflexes are recognized as major causes of aspiration pneumonia in older individuals (170). A high-quality review confirmed that aging weakens respiratory and swallowing muscles and causes sarcopenia in multiple ways; thus, older adults with CAP may be more challenging (171). Sarcopenia is an aging-related change in which skeletal muscle mass decreases, skeletal muscle strength decreases, and physical function decreases with age (172). The results of a meta-analysis indicate that sarcopenia is highly prevalent in individuals with respiratory disease (173). The onset of sarcopenia is closely linked to inflammation and immune senescence and eventually leads to frailty (Figure 5).

Inflammation leads to muscle proteolysis by affecting elevated levels of TNF-α, IL-6, IL-1, and CRP and inhibits protein synthesis through activation of the NF-κB and ubiquitin–proteasome pathways, which affects the balance between anabolic and catabolic processes in muscle, resulting in sarcopenia (174). A study has postulated that muscle injury in an older person combined with immune aging would cause secondary damage to healthy muscle from aberrantly migrating neutrophils, resulting in myocyte damage and apoptosis and loss of muscle fibers, which could result in functional loss and physical weakness associated with frailty (175). Frailty is an extremely relevant geriatric condition in an aging society, and its occurrence increases with age (176). It can be defined simply as the clinical state in which an individual increases vulnerability to the development of negative health-related events when exposed to endogenous or exogenous stressors (177). It can be viewed as a progressive age-related decline in physiological systems, leading to a reduction in intrinsic capacity reserves, making people highly vulnerable to stress and increasing the risk of a range of adverse health outcomes (178). Sarcopenia is a high risk factor for frailty, leading to increased mortality (172). Frailty syndromes might result in the following: poorer response to somatic and/or psychiatric stressors, increased risk of hospitalization, adverse disease outcomes, hospitalization, and premature death (179). Hence, both sarcopenia and frailty are extremely challenging for older adults with CAP. Previous studies have revealed relationships among oxidative stress, sarcopenia and frailty (52, 180). In addition to the previously mentioned immune senescence- and inflammation-related mechanisms, sarcopenia is also associated with oxidative stress in skeletal muscle. An animal study found that mice deficient in Cu/ZnSOD exhibited sarcopenia (181). Oxidative stress promotes muscle atrophy, so skeletal muscle atrophy can be treated by targeting antioxidative stress (182). For example, an early animal study noted that VitE and C supplementation reduced oxidative stress and improved antioxidant enzymes and positive muscle work in chronically loaded muscles of aged rats (105). There are also studies summarizing the preventive and therapeutic potential of a particular type of VitE, tocotrienol, whose superior antioxidant capacity improves antioxidant defense mechanisms in muscle-forming cells, enhances musculoskeletal health and addresses age-related musculoskeletal problems (102, 183). According to a recent study, melatonin targets mitochondria, scavenges free radicals and reduces oxidative damage, with a potential and promising therapeutic role in limiting muscle degeneration (mainly mitochondrial function) and sarcopenia (184). This conclusion has also been confirmed in a recent animal study. Following melatonin treatment, histological damage was alleviated, and the cross-sectional area of muscle fibers in the gastrocnemius tissues of middle-aged mice increased. Furthermore, the percentage and size of normal mitochondria increased, while the levels of MDA and ROS decreased in these tissues (119).

Previous studies have suggested that oxidative stress may be associated with frailty by affecting RNA oxidation, absolute telomere length shortening, increased markers of oxidative stress, and mitochondrial dysfunction and it was also found that the deleterious effects of both oxidative stress and frailty increase linearly with aging (185, 186). Therefore, it may be feasible to intervene in frailty by modulating oxidative stress. An early clinical trial involving 827 older adults revealed an association between low circulating levels of VitE, one of the most important components of the human antioxidant system, and the presence of frailty. Participants with the highest VitE levels were less likely to be frail than those with the lowest VitE levels were (103). The results from a 12-year Framingham Heart Follow-Up Study suggest that higher dietary quercetin intake is particularly strongly associated with lower odds of frailty onset in adults (187).

Surely, the onset and development of sarcopenia and frailty are not determined by only oxidative stress, and oxidative stress is only one of the important mechanisms. However, relevant studies have been able to affirm, to some extent, the positive significance of modulating oxidative stress in sarcopenia and frailty, which could be instructive for the future of older CAP patients with related diseases.

### 5.5 Dealing with aging, multimorbidity and polypharmacy in the older population

Aging is characterized by a progressive loss of tissue and organ function caused by genetic and environmental factors, nutrition and lifestyle. Oxidative stress is one of the most important mechanisms of increased cellular senescence and frailty, leading to a wide range of diseases that are common in the older population, including chronic lung disease, cardiovascular disease, chronic kidney disease, skeletal muscle dysfunction and cancer (188, 189). In such cases, a public health problem arises—multimorbidity—in which individuals develop two or more chronic pathologies. It is estimated that more than half of the population over the age of 60 is affected (190). The requirement of medications as first-line treatment for these chronic conditions increases the potential cost of treatment, leaving older adults in a polypharmacy dilemma and causing many negative effects. It is also directly correlated with the development of frailty, which may further compromise the antioxidant defense system (191). Risk factors such as malnutrition, multimorbidity and polypharmacy also increase the risk of severe illness and death in older CAP patients (192). In cases where oxidative stress is the pathophysiological basis, some natural antioxidant products work through different mechanisms than conventional drugs do to enhance therapeutic efficacy, avoiding the need to add new drugs to basal therapies, thus achieving therapeutic goals at lower doses of standard therapies (191). One study revealed that the use of vitamins C and/or E in men taking daily metformin improved insulin resistance and fasting blood glucose levels, perhaps without the need to add another hypoglycemic agent (106). A meta-analysis revealed that VitC supplementation in patients with chronic obstructive pulmonary disease (COPD) increased serum antioxidant levels (VitC and GSH) and improved lung function [FEV1% and FEV1/FVC; (193)]. Another review discussed the beneficial health effects of VitE in the prevention or treatment of hyperlipidemia, diabetes, osteoporosis, and cancer (194). Quercetin can be a potential treatment agent for a variety of inflammatory conditions, and it can also lower blood pressure, lower cholesterol levels, improve endothelial function, and even exhibit anticancer effects by inhibiting cancer cell proliferation and inducing apoptosis (195). Some reviews affirmed the importance of the antioxidant effects of melatonin in ameliorating chronic kidney disease and diabetic chronic kidney disease and proposed that endogenous antioxidants have fewer side effects than do exogenous antioxidants (196, 197). CoQ10 supplementation has a positive effect on mitochondrial deficiency syndrome and some of the symptoms of aging, whereas cardiovascular disease and inflammation are mitigated by the antioxidant effects of CoQ10 (198).

Additionally, some studies have indicated that certain medications for underlying conditions, commonly used in older adults, can lead to nutrient deficiencies. For example, individuals with hyperlipidemia treated with statins exhibit significant reductions in CoQ10, α-tocopherol, and other important antioxidants. These reductions are thought to drive statin-induced myopathy (199). Coadministration of CoQ10 may ameliorate these effects, but additional studies are needed to confirm this, particularly in older adults experiencing or at risk of polypharmacy (200). Long-term, high-dose aspirin use has been associated with decreased VitC levels, which can lead to gastric mucosal thinning, gastritis, peptic ulcer disease, nausea, anorexia, and malnutrition (199, 201). However, the doses used for primary and secondary cardiovascular disease prophylaxis are much lower. Moreover, there is currently no evidence suggesting a similar reduction in VitC levels or a need for VitC supplementation in chronic low-dose aspirin users (200).

In fact, patients with CAP cannot avoid the risk of multimorbidity and polypharmacy, and some chronic diseases are even detected during the treatment of CAP. Owing to various changes in the modern environment, diet and life structure, this situation can hardly be solved at the source and plagues the treatment of many diseases, not only CAP. Multiple medications may interact with each other and, to some extent, may be detrimental to the treatment of CAP or even affect the treatment plan for CAP. However, oxidative stress has been linked to many diseases in older individuals, and it might be a good attempt to intervene in chronic diseases from the perspective of modulating oxidative stress. In particular, many natural antioxidant compounds have relatively safe compositions and a wide therapeutic range, and we are confident in their further development and use in the future.

## 6 Impact of COVID-19

Since the worldwide outbreak of SARS-CoV-2, oxidative stress has attracted a great deal of attention, and there has been a proliferation of scientific studies on the theory and practice of its relationship with pneumonia (202, 203). As more is known about the disease and a vaccine has been developed, the threat of COVID-19 is diminishing, but some patients experience persistent symptoms or sequelae even after recovering from COVID-19 (204, 205). The estimated overall global prevalence of COVID-19 sequelae is approximately 43%, with major clinical manifestations, including pulmonary, cardiovascular, renal, neuropsychiatric, and other related symptoms (206). Moreover, COVID-19 appears to have long-term health effects, which may put strain on the health care system. This long-term COVID-19 affects a large proportion of people in all age groups, and older adults are characterized by age-specific manifestations and are more likely to have persistent symptoms or sequelae, increasing susceptibility to the disease and affecting quality of life (207). A Korean study revealed that COVID-19 negatively affects physical activity, quality of life, and memory in older adults (> 55 years old), leading to mental diseases such as insomnia and depression (208). Therefore, after the onset of COVID-19, older CAP patients deserve more attention, and providing more comprehensive therapeutic measures and modulating the oxidative stress state may be a good choice.

For example, melatonin reduces the long-term inflammatory and oxidative effects of viruses, stimulates the suppressed immune system, and has the potential to ameliorate inflammation and inhibit the cytokine storm induced by SARS-CoV-2 (209). Treatment with antioxidant supplements such as NAC, VitC, VitE, and melatonin in COVID-19 patients reduces biomarkers of oxidative stress and inflammation, such as total systemic antioxidant capacity, CRP, IL-6, and lipid peroxidation, and improves the survival score (210). However, a recent study of markers of oxidative stress in older adults with SARS-CoV-2 infection revealed that oxidative stress was associated with fatal events as well as near-term prognosis in older adults with SARS-CoV-2 infection but was less important than other predictors (e.g., cardiovascular factors) of long-term prognosis at 2 years (211). Notably, the unique active ingredients in various natural traditional Chinese medicine (TCM) herbs also play important roles in the prevention and treatment of COVID-19 through their anti-inflammatory, antioxidant and antiapoptotic effects (212). In fact, the respiratory tract and lungs bear the brunt of this worldwide epidemic. Even if most older adults are clinically cured, the respiratory damage caused by COVID-19 should not be ignored. In conjunction with existing research, modulating oxidative stress homeostasis can indeed benefit older adults to some extent and increase their resistance to future threats of challenges such as COVID-19.

## 7 Limitations and challenges of modulating oxidative stress in older CAP patients

Oxidative stress has been demonstrated to be associated with CAP in older individuals through various theories and practices. Free radicals act as physiological signaling messengers to oxidatively modify biologically essential molecules to maintain normal cellular physiology and pathology. The current concern is that excessive or inappropriate antioxidant interventions in aging CAP instead disrupt normal cell signaling pathways and inhibit the beneficial effects of ROS and lipid peroxidation products on disease, such as by mediating the physiological apoptosis of immune cells (213). This, in turn, deviates from the original intent of antioxidant therapy and negatively affects inflammation, the immune response, etc., driving the progression of CAP and increasing the risk of CAP. In particular, older people also have chronic low-grade inflammation, so antioxidants should be carefully applied. Moreover, not all conditions are suitable for antioxidative stress treatment. Researches have indicated that some pathogens respond to host oxidative stress by producing GSH to survive in the environment and the host. This mechanism involves most gram-negative bacteria and a few gram-positive bacteria and is more prevalent and essential in eukaryotic cells (214). Experiments have also demonstrated that GSH biosynthesis-deficient *Pseudomonas aeruginosa* mutants are less virulent in an acute pneumonia infection model (215). Therefore, analyzing from the other side, increasing the oxidative stress response of the cells can serve to deplete part of the GSH defense produced by the pathogen, thus reducing its virulence. It has also been suggested that the development of chronic bacterial infections and antibiotic resistance is associated with altered adaptation of pathogens to oxidative stress, suggesting that chronic infections and antibiotic resistance can be counteracted by increasing oxidative stress in cells (67). However, controlling the degree of enhancement to inhibit the pathogen while avoiding excessive oxidative stress in the organism is still a current challenge.

Studies show that antioxidants can have dichotomous roles in ROS production. They are easily oxidized and can act as oxidants to induce damage at high concentrations (100). An early animal study demonstrated that VitC primarily acts as an antioxidant at low doses, whereas pro-oxidant effects predominate at high doses. Thus, its use at pharmacological doses should be approached with caution (95). Such differences are also observed in clinical trials. For example, the effects of different high doses of VitC are not uniform in patients with severe COVID-19 (96). Administering 3 g/day of VitC to moderate and severe COVID-19 ICU patients reduced inflammation and mortality without adverse effects. However, a dose of 6 g/day has no significant impact on ICU length of stay or mortality (97). Earlier studies have shown that in healthy volunteers, daily doses of VitC exceeding 500 mg tend to decrease its bioavailability. Safe daily doses are generally less than 1000 mg, and doses above 400 mg daily offer no additional benefit (216). However, the optimal dose of VitC that is both safe and effective for antioxidant activity in disease states remains unclear. In addition, studies have shown that NAC can protect melanocytes from oxidative stress and damage and delay the onset of ultraviolet-induced melanoma in mice. However, high doses may increase Nrf2 nuclear translocation, potentially promoting metastasis (217, 218).

In summary, we advocate the healthy modulation of oxidative stress in older CAP patients to achieve the desired therapeutic assistance rather than simply inhibiting and scavenging free radicals. However, although common antioxidants such as vitamins, NAC, and Co Q10 are commonly used as drugs in the clinic, the emphasis is still on their primary pharmacological effects, and their antioxidant effects remain at the research stage. The wide variety of antioxidants that have been identified are constantly being depleted, as they work with free radicals, and different antioxidants are often accompanied by other biological effects. Therefore, the choice of antioxidants to be used to treat older adults with CAP, the dosage and timing of their use, and the optimal mode of administration are still not very clear, and more targeted studies are needed.

Given existing studies, we propose focusing research on adjunctive treatments for CAP with combined antibiotics to alleviate clinical symptoms while considering the unique characteristics of older adult patients. Most studies have focused on COVID-19 patients, with fewer addressing CAP caused by other pathogens, limiting generalizability. To avoid drug interactions, the study population should initially consist of older CAP patients without comorbidities, then expand to include those with one or more comorbidities, malnutrition, or immunodeficiencies. Drug selection should prioritize single-agent therapies with high safety profiles, clear mechanisms of action, high bioavailability, and suitability for CAP treatment, such as NAC. Determining drug dosage, timing, and mode of administration should begin with *in vitro* experiments, reference clinically established safe dosages, and integrate drug toxicology. Oxidative stress involves multiple signaling molecules and reactive enzymes; thus, detection indices should be both experimentally valuable and universally applicable. Incorporating the effects of pathogenic microorganisms, inflammation, and immune responses into the study design would further enrich the experimental content.

## 8 Conclusion

Global aging has resulted in an enormous increase in the population of older people and an enormous challenge to global public health. The impact of CAP, one of the most common respiratory system diseases, on the older population is obvious. The onset and development of CAP are closely related to several pathophysiological aspects, including pathogenic microorganisms, inflammation, immunity, and oxidative stress. In older CAP patients, there are also multiple age-related risk factors, such as immune senescence, sarcopenia, and polypharmacy, that influence the clinical presentation, development, and prognosis. Anti-infective therapy is still the primary treatment for CAP, but inappropriate use of antibiotics is common in older individuals, and older individuals are particularly susceptible to antibiotic-associated adverse effects. Modulating the signaling pathways that cause the inflammatory response and improve the immune function of patients has become the focus of reducing inflammatory damage in the lungs, especially in older adults with CAP. Oxidative stress, as an important mechanism of CAP, has been widely demonstrated to be related to inflammation and immunity. Especially after the occurrence of COVID-19, a number of studies related to oxidative stress have been conducted, but they tend to be limited to inflammation and immunity only, ignoring the possible additional links with the older population, who are the most infected with CAP. Therefore, we attempted to further clarify the link between oxidative stress and age-related risk factors such as immune senescence, sarcopenia, frailty, aging, and multimorbidity in the context of older adults CAP patients, as well as the greater potential value and prospects of modulating oxidative stress in treating older adults with CAP.

Clearly, there is no doubt that targeted modulation of oxidative stress benefits older adults CAP patients. However, many challenges and unknowns concerning how to modulate oxidative stress for further practical clinical applications exist, and more targeted research is needed. This review is limited in length, and we mainly discuss toward the characteristics of older adults CAP, emphasizing the value of modulating oxidative stress; thus, we only briefly describe the various immune, inflammatory, and aging mechanisms and only cover the study of common antioxidants, such as GSH, NAC, vitamins, CoQ10, and melatonin. If readers need further information, please refer to the outstanding articles cited in the text for relevant content.
